# Development and initial pilot validation of a treatment fidelity instrument for family-based interoceptive exposure for adolescents with low-weight eating disorders

**DOI:** 10.1371/journal.pone.0288125

**Published:** 2023-07-06

**Authors:** Deena Peyser, Kayla Costello, Robyn Sysko, Kurt Schulz, Tom Hildebrandt

**Affiliations:** Eating and Weight Disorders Program, Department of Psychiatry, Icahn School of Medicine at Mount Sinai, New York, NY, United States of America; University of Gothenburg, SWEDEN

## Abstract

**Background:**

This pilot study outlines the development and psychometric evaluation of a therapist adherence coding measure for a novel treatment, Family-Based Treatment Interoceptive Exposure (FBT-IE).

**Methods:**

The IE Adherence Coding Framework (IE-ACF) was developed from the FBT-IE Manual using an iterative process. Items on the IE-ACF were coded by two independent coders as either present or absent with therapists considered adherent if both independent coders coded the item as “present.” Videotaped sessions of FBT-IE of 30 adolescents with low-weight eating disorders (DSM-5 typical/atypical anorexia nervosa) and their families were coded. Participants received the FBT-IE intervention as part of a randomized controlled trial.

**Results:**

Seventy FBT-IE videos were coded. The IE-ACF identified a mean (SD) rating of 80% (±5%) therapist adherence to the protocol across the six-session treatment, with a per item adherence ranging from 36–100%. Two independent coders demonstrated moderate to almost perfect inter-rater reliability (κ range 0.78–0.96) across the sessions.

**Conclusion:**

IE-ACF measured therapist adherence to our novel FBT-IE treatment for adolescents with low-weight eating disorders. Through this study, we demonstrated that 1) our therapists were adherent to the FBT-IE manual in the context of an ongoing clinical trial and 2) that independent coders reliably coded sessions using our novel IE-ACF.

## Introduction

Therapist fidelity measures are intended to examine the degree of adherence to an intervention and are essential to ensure that evidence-based treatments are delivered as intended. Adherence to treatment refers to the extent to which a therapist delivers the intervention with integrity and according to the procedures outlined by the manual [1]. Therapist adherence is crucial for successful implementation and evaluation of an intervention and to ensure that the intervention can be replicated reliably [2, 3]. It is particularly important in randomized controlled trials, not only to monitor the extent to which treatment is implemented consistently, but also to ensure differentiation in the delivery of distinct treatment interventions [4, 5].

Family-based therapy (FBT) is the leading evidenced based treatment for adolescents with anorexia nervosa (AN). However, not all patients recover using this treatment with low sustained remission rates (e.g. 41%; [6]) at one year among those treated with FBT, suggesting that it is challenging to maintain treatment gains. As part of a larger project intended to modify the effective connectivity of insula-amygdala-ventral striatum neurocircuitry, we modified the comprehensive Exposure-Based Family Therapy (FBT-E) to include a Family-Based Treatment Interoceptive Exposure (FBT-IE) protocol for adolescents with low-weight eating disorders to address aspects of this potentially chronic condition that are not primary targets of other treatments. FBT-IE is based on associative learning principles and directly targets disgust sensations evoked by food and feeling of fullness that lead to avoidance. This treatment is distinct from traditional FBT, which is an agnostic contingency management-based intervention that aims to change the incentive value of food through weight gain and parent empowerment. Furthermore, in FBT-IE, parents function more as ‘coaches’ in facilitating and tolerating exposures to challenging food stimuli, rather than acting as the ‘inpatient staff,’ or the primary decision makers, during the refeeding process, consistent with classic FBT [7].

Our novel six-session FBT-IE treatment is based on the ‘broad anxiety’ model of AN [8] and is designed to modify disgust. This intervention targets the disgust conditioning model and the brain neurocircuitry hypothesized to contribute to pathological food avoidance in low-weight eating disorders (DSM-5 typical/atypical anorexia nervosa). During the FBT-IE intervention, the therapist teaches skills, such as mindfulness to tolerate distress to visceral sensations evoked by food and eating [9] that lead to avoidance through associative learning. During each session, patients, with their parents’ support, participate in an in-vivo exposure exercise in which the family is provided with a meal replacement shake of unknown content and caloric density. During the exposure exercise, the patient practices applying the skills taught during the session with the goal of utilizing and applying these skills with other challenging and restricted foods outside of session. The goal of delivering this FBT-IE intervention to adolescents with low-weight eating disorders was to determine if this intervention has a unique impact on food avoidance, independent of weight gain.

We developed a treatment manual outlining the six intervention sessions, which the therapists reviewed and followed for the research study. In conjunction, we developed an Adherence Coding Framework (See S1 Appendix), which includes dichotomous ratings for each of the session targets. This tool enables independent coders to evaluate and measure treatment fidelity accurately. It is critical, yet often overlooked, to establish a means to evaluate the implementation of an intervention and to ensure that a treatment is delivered consistently and reliably according to the intended elements. While treatments for a range of psychological disorders in adolescents, including depression and substance use disorders, have reported on adherence (e.g. [10–12] only a few report therapist treatment fidelity for adolescents with eating disorders (e.g. [13–15]). Furthermore, only one of these studies [15] provides the coding framework so that mental health professionals implementing the treatment can evaluate treatment fidelity to FBT using a validated measure. As such, we believe that the development of the IE Adherence Coding Framework (IE-ACF) and the evaluation of its psychometric properties will facilitate appropriate implementation and evaluation of this novel treatment.

Thus, the aim of this paper is to describe the development and psychometric validation of a treatment fidelity instrument, the IE-ACF, for adolescents with low-weight eating disorders. Data for this study were collected from an ongoing randomized controlled trial testing a short form of FBT-IE based on the ‘broad anxiety’ model of AN [8, 9, 16]. Many individuals with low-weight eating disorders experience a visceral disgust response to palatable food in addition to a fear of gaining weight. We hypothesized that disgust may contribute to food avoidance among adolescents with low-weight eating disorders through hypersensitivity to interoceptive experiences of eating and feelings of fullness. This disgust response, unlike fear, is resistant to extinction making it a unique treatment target that may be more responsive to FBT-IE, which focuses on learning to tolerate the discomfort associated with food and eating [9].

## Methods

### Study design and participant characteristics

This six-session FBT-IE intervention served as a proof of concept with the intention of ensuring reliability. The sample for this study included videotaped sessions of 30 adolescents, ages 12–18, with low-weight eating disorders (defined as DSM-5 typical/atypical anorexia nervosa) and their parent(s) who were randomized to FBT-IE.

### Ethics approval and consent to participate

The study was reviewed and approved by the institutional review board of the hospital in which the study took place. Adult participants and parents provided written consent and adolescents provided written assent.

### Therapist training and adherence to treatment protocol

Four therapists delivered the FBT-IE treatment. Therapists were either licensed psychologists or post-doctoral fellows with eating disorder expertise who were trained to administer the manual based FBT-IE treatment. Forty-nine percent of FBT-IE therapy sessions were recorded and coded for treatment fidelity according to the IE-ACF (See S1 Appendix). Each video was coded by two independent coders.

### Fidelity measure

The development of the IE-ACF was an iterative and collaborative process with the senior author (T.H.) creating a coding framework based on the FBT-IE Manual. Items were then reviewed with a team of coders comprised of researchers, clinical psychologists, postdoctoral fellows and graduate school externs. Items were reviewed for clarity, redundancy, and accurate representation of items in the manual. Subsequently, selected videotaped therapy sessions were viewed by the independent coders and reviewed in a group format to discuss challenges, discrepancies, and consensus on particular items. The coding framework was then revised as needed until the IE-ACF was finalized.

The IE-ACF developed from this process is a checklist that includes 12–15 items for each of the six therapy sessions (Table 1). Items on the coding sheets vary based on the study session and the interventions used during that session with adherence rated for the presence (“1”) or absence (“0”) of treatment intervention targets, and not competence, in administering the intervention. The majority of the items relate to therapist behaviors and implementation of the treatment. However, because parents are actively involved in the treatment, several items ask about parent behavior as they are relevant to measuring family-based interventions and data suggest that parental self-efficacy contributes to better treatment outcomes in adolescents with anorexia nervosa [17].

**Table 1:** FBT-IE adherence checklist items for treatment Sessions 1–6.

Item	Session 1	Session 2	Session 3	Session 4	Session 5	Session 6
Did the therapist weigh the patient?	X	X	X	X	X	X
Did the therapist introduce study and give an overview of treatment structure?	X					
Did the therapist provide psycho-education on the weekly skill?	X	X	X	X	X	X
Did therapist give rationale for exposure (facing aversive internal experiences)?	X					
Did the therapist clarify roles of parents and siblings in treatment?	X					
Did the therapist create somatic symptom hierarchy?	X					
Did the therapist introduce and describe the aims of the Interoceptive Exposure Exercise (shake)?	X					
Did the therapist review the role of counter-conditioning?	X					
Did the therapist provide the shake?	X	X	X	X	X	X
Did the therapist provide a competent execution of exposure task?	X	X	X	X	X	X
Did the therapist encourage non-judgmental labeling of somatic symptoms during the IE exercise?	X	X	X	X	X	X
Did therapist encourage parents to adopt role as ‘coach’ during the IE exercise?	X	X	X	X	X	X
Did parents encourage/push child to drink more of shake during the IE exercise?	X	X	X	X	X	X
Did therapist meet alone w/ parents for 10 minutes?	X	X	X	X	X	
Did parents express lack of confidence in ability to implement tx? (“I can’t do this”)	X	X	X	X	X	X
Did the therapist review the previous week (IE challenge and counterconditioning)?		X	X	X	X	X
Did the therapist encourage patient to utilize weekly skills during the IE exercise?		X	X	X	X	X
Did the therapist review the weekly skill and how to apply it to the IE challenge food tasks throughout upcoming week?		X	X	X	X	
Did the therapist complete the ‘eating a raisin mindfully’ exercise?		X				
Did the therapist complete the mindful and controlled breathing exercises?			X			
Did the therapist complete the finger-trap exercise?				X		
Did the therapist complete the short-term positive-coping activities exercise?					X	
Did the therapist complete the acceptance metaphor exercises?						X
Did the therapist summarize the main points of treatment and review upcoming sessions following the end of the intervention?						X

X indicates that intervention should occur during the session based on the FBT-IE Manual.

^a^FBT-IE = Family Based Therapy Interoceptive Exposure

^b^IE = Interoceptive Exposure

^c^Tx = treatment

### Training of raters

The eight coders included a post-doctoral psychology fellow specializing in eating disorders and trained in delivering the treatment intervention, bachelor and masters level research assistants and undergraduate volunteers working in an eating disorders clinical research setting. All coders were considered fully trained in the coding process after undergoing training with T.H. or D.P., which included didactics, role plays, and reviewing therapy videos from all six sessions of FBT-IE over the course of several weeks. All coders attended ongoing supervision throughout the duration of the coding period.

### Statistical analyses

Therapists were considered adherent to each item if both independent coders marked them as adherent (e.g. 1). Inter-rater reliability was examined by calculating kappa scores for each of the six treatment sessions. Kappa values range from -1.0 to 1.0 with the latter indicating perfect reliability [18]. Kappa scores between 0.60 and 0.79 were considered moderate, between 0.80 and 0.90 strong, and above 0.90 almost perfect [19].

## Results

### Descriptive statistics

Table 1 outlines the adherence coding items for sessions 1–6. Table 2 depicts adherence per item for each of the six therapy sessions. Adherence varied from 36% to 100% on individual items in each session. Mean (SD) therapist adherence across the six sessions was 80% (±5%). Table 3 shows inter-rater reliability for each of the six treatment sessions. The kappa values for each of the six therapy sessions were 0.93, 0.96, 0.83, 0.78, 0.85, 0.83, respectively, consistent with moderate to almost perfect inter-rater reliability.

**Table 2:** Adherence To session treatment items.

	Adherence (%)					
Item	Session	Session	Session	Session	Session	Session
	1	2	3	4	5	6
Weight collected*	100	91	92	100	100	100
Study overview	91					
Explain 3 aspects of anxiety	100					
Introduce weekly skill		100	100	100	100	90
Rationale for exposure	100					
Clarify family roles	100					
Create somatic symptom hierarchy	64					
Describe aims for IE exposure (shake)	100					
Review counter-conditioning	91					
Provide shake	100	100	100	100	100	100
Competent execution of exposure	100	100	100	100	91	100
Encourage symptom labeling	100	90	64	64	40	70
Therapists encouraged parents as coach	45	50	55	36	45	50
Parents encourage child to drink shake	64	50	55	50	55	70
Therapist & parents met alone	91	78	64	100	56	
Parents express lack of confidence**	82	100	82	82	89	78
Review prior week challenge		36	62	83	67	60
Encourage skill application during IE		100	82	75	70	40
Explain how to use skill during the week		100	73	45	70	
Raisin Exercise		100				
Mindful Breathing Exercise			100			
Finger-Trap Exercise				67		
Positive Coping Exercise					100	
Acceptance Exercise						90
Reviewed all skills taught & next steps						60
Mean adherence without ‘coach’***	92	87	81	80	78	78
Mean Overall Adherence	88	84	79	77	76	76

IE = Interoceptive Exposure.

*Weights were at times gathered off-camera, but chart review confirmed all patients were weighed each week except for one patient during one session

**Items were reverse coded

***Mean adherence without ‘therapist encouraged parent as coach’ item

**Table 3:** Kappa scores for FBT-IE treatment sessions.

Kappa					
Session 1	Session 2	Session 3	Session 4	Session 5	Session 6
0.93	0.96	0.83	0.78	0.85	0.83

FBT-IE = Family Based Therapy Interoceptive Exposure.

### Discussion

This pilot study is the first to develop and validate an adherence coding measure (the IE-ACF) to evaluate treatment fidelity to a novel FBT-IE treatment for adolescents with low weight eating disorders. The data demonstrated that 1) six sessions of FBT-IE can be delivered with adherence to the treatment manual in the context of an ongoing clinical trial and 2) that independent coders reliably coded sessions as adherent using our novel adherence coding tool.

Inter-rater reliability was moderate to almost perfect across the study sessions (κ range 0.77–0.96), which suggests that IE-ACF is a reliable measure for evaluating therapist adherence to the FBT-IE treatment. Therapist adherence to the manual was present across the sessions (range 76–88%), while adherence to individual session items varied considerably (range 36%-100%). Across sessions, Sessions 1 and 2 demonstrated the highest inter-rater reliability, which we attribute to greater clarity in the coding items for these sessions. Two broad features of the IE-ACF likely contributed to the high levels of adherence and inter-rater reliability, namely the use of a treatment manual and binary coding. Treatment manuals reduce the variability in session content, promote treatment fidelity, and facilitate the training of therapists, ongoing supervision, and the training of adherence coders. The use of a binary coding scheme facilitated the use of objective assessments and promotes inter-rater reliability.

To examine more specific factors that may have affected our outcomes, the lowest scoring items were reviewed to understand reasons for variability in adherence. The item “Did parents encourage/push child to drink more of shake during the IE exercise?” was consistently low (range: 50–70% adherence) as it proved challenging for the parents to push their child to consume the shake amidst resistance, uncertainty, and the expression of disgust. The item “Did therapist encourage parents to adopt role as ‘coach’ during the IE exercise?” was also consistently low across sessions. In delivering this treatment intervention, the therapist only used the term “coach” when the sports analogy was salient and relevant to the family. While therapists generally adhered to the treatment framework, they approached the intervention flexibly acknowledging that each family is unique and not all families identified with every metaphor. This balance of structure and flexibility reflects the general approach to the delivery of manual-based treatment in the field more broadly. We believe this item would have demonstrated greater adherence if the item assessed whether the therapist encouraged the parents to assume a supportive or authoritative role. We, therefore, calculated mean adherence per session with and without this item included (Table 2).

Variability in adherence to other treatment items can be attributed to different approaches by the therapist in administering therapeutic content or different interpretations by the coder in coding the items. Objective items such as “did the therapist provide the shake” had higher adherence than less objective items such as the therapists’ explanation of how to apply a skill during the week. There were also practical elements that impacted adherence, such as the therapist not having a finger trap available during the session that included the exercise utilizing it.

The findings of this study should be considered in light of the primary limitation of sample size. Only 49% of total available sessions were videotaped, which may have introduced a selection bias attributable to random recording loss due to sound quality, extracting data, or uploading videos. However, all videotaped sessions with adequate audio quality were coded, which reduces the chance of a systematic bias. Additionally, data from the IE-ACF are consistent with others in the field. Perfect inter-rater reliability scores are rare, and previous studies in FBT report a wide range from poor to almost perfect inter-rater reliability scores for ratings of treatment fidelity [15]. Finally, as with many measures of treatment fidelity, the described therapist adherence measure focuses on prescribed treatment components but not proscribed aspects. Adherence measures would benefit from including both prescribed and proscribed treatment elements to ensure delivery of distinct treatment interventions [5]. Future research in this area would be beneficial. Despite these limitations, this study has multiple strengths. This is the first study to present a treatment fidelity measure for FBT-IE, to assess adherence to this therapy, and to demonstrate strong adherence and reliability to our novel treatment intervention.

In summary, the psychometric properties of IE-ACF showed 80% adherence of therapists to the treatment manual and moderate and almost perfect inter-rater reliability in the coding of adherence by two trained, independent coders. Future research should examine the relationship between therapist adherence to FBT-IE and treatment outcomes.

## Supporting information

S1 Appendix Interoceptive exposure-adherence coding framework (IE-ACF). (DOCX)

## Abbreviations

FBT-E: Exposure-Based Family Therapy
FBT: Family-based therapy
FBT-IE: Family-Based Treatment Interoceptive Exposure, IE = Interoceptive Exposure
IE-ACF: Interoceptive Exposure Adherence Coding Framework
SD: Standard Deviation
Tx: Treatment
