# Supplementary material for: Development and initial pilot validation of a treatment fidelity instrument for family-based interoceptive exposure for adolescents with low-weight eating disorders
Source: PLoS One. 2023 Jul 6;18(7):e0288125. doi: 10.1371/journal.pone.0288125 (PMC10325038; doi:10.1371/journal.pone.0288125)
Supplement: S1 Appendix — (DOCX) [file pone.0288125.s001.docx]

ID#:_____ SESSION#:_____ LENGTH:_____ THERAPIST INITIALS:_____ RATER:_____

**INTEROCEPTIVE EXPOSURE: SESSION ONE**

1. ______Did the therapist weigh the patient?
2. ______Did the therapist introduce study and give an overview of treatment structure?
3. ______Did the therapist provide psycho-education on three components of anxiety?
   1. fear
   2. worry
   3. disgust
4. ______Did therapist give rationale for exposure (facing aversive internal experiences)?
5. ______Did the therapist clarify roles of parents and siblings in treatment?
6. ______Did the therapist create somatic symptom hierarchy with columns for:
   1. somatic symptoms
   2. foods associated with somatic symptoms
   3. Levels of disgust
7. ______Did the therapist introduce and describe the aims of the Interoceptive Exposure Exercise (shake)?
8. ______Did the therapist review the role of counter-conditioning?
9. ______Did the therapist provide the shake?
10. ______Did the therapist provide a competent execution of exposure task via one of the following:
    1. prompting patient to increase disgust consistently throughout exposure task
    2. eliciting self-reported disgust by patient
    3. using motivational strategies to encourage patient to increase intake of shake, thereby achieving a higher level of disgust
11. ______Did the therapist encourage non-judgmental labeling of somatic symptoms during the IE exercise?
12. ______Did therapist encourage parents to adopt role as ‘coach’ during the IE exercise?
13. *______Did parents encourage/push child to drink more of shake during the IE exercise?
14. ______Did therapist meet alone w/ parents for 10 minutes and hand out parent manual?
15. *____Did parents express lack of confidence in ability to implement tx? (“I can’t do this”)

ID#:_____ SESSION#:_____ LENGTH:_____ THERAPIST INITIALS:_____ RATER:_____

**INTEROCEPTIVE EXPOSURE: SESSION TWO**

1. ______Did the therapist weigh the patient?
2. ______Did the therapist review the previous week?
   1. IE challenge food tasks completed during week
   2. Attempts at counter-conditioning during week?
3. ______Did the therapist provide psycho-education on weekly skill: mindfulness and it’s following components:
   1. observing
   2. describing
   3. judgmental vs. non-judgmental language
4. ______Did the therapist complete the ‘eating a raisin mindfully’ exercise?
5. ______Did the therapist provide the shake?
6. ______Did the therapist provide a competent execution of exposure task via one of the following:
   1. prompting patient to increase disgust consistently throughout exposure task
   2. eliciting self-reported disgust by patient
   3. using motivational strategies to encourage patient to increase intake of shake, thereby achieving a higher level of disgust
7. ______Did therapist encourage non-judgmental labeling of somatic symptoms during IE exercise?
8. ______Did the therapist encourage parents to adopt role as ‘coach’ during IE exercise?
9. ______Did the therapist encourage patient to utilize weekly skills (observing and non-judgmentally describing) during the IE exercise?
10. ______Did the therapist review the weekly skill and how to apply it to IE challenge food tasks throughout upcoming week?
11. *______Did parents encourage/push child to drink more of shake during the IE exercise?
12. _____Did therapist meet alone w/parents for 10 mins to problem-solve upcoming week?
13. *____Did parents express lack of confidence in ability to implement tx? (“I can’t do this”)

ID#:_____ SESSION#:_____ LENGTH:_____ THERAPIST INITIALS:_____ RATER:_____

**INTEROCEPTIVE EXPOSURE: SESSION THREE**

1. ______Did the therapist weigh the patient?
2. ______Did the therapist review the previous week?
   1. IE challenge food tasks completed during week
   2. Attempts at counter-conditioning during week?
3. ______Did the therapist provide psycho-education on weekly skill: increasing flexibility and reducing attempted control over natural bodily functions, such as breathing and eating?
4. ______Did the therapist complete the mindful and controlled breathing exercises?
5. ______Did the therapist provide the shake?
6. ______Did the therapist provide a competent execution of exposure task via one of the following:
   1. prompting patient to increase disgust consistently throughout exposure task
   2. eliciting self-reported disgust by patient
   3. using motivational strategies to encourage patient to increase intake of shake, thereby achieving a higher level of disgust
7. ______Did the therapist encourage non-judgmental labeling of somatic symptoms during the IE exercise?
8. ______Did therapist encourage parents to adopt role as ‘coach’ during the IE exercise?
9. ______Did the therapist encourage patient to utilize weekly skills (reduce rigid control) during the IE exercise?
10. ______Did the therapist review the weekly skill and how to apply it to IE challenge food tasks throughout upcoming week?
11. *______Did parents encourage/push child to drink more of shake during the IE exercise?
12. _____Did therapist meet alone w/parents for 10 mins to problem-solve upcoming week?
13. *____Did parents express lack of confidence in ability to implement tx? (“I can’t do this”)

ID#:_____ SESSION#:_____ LENGTH:_____ THERAPIST INITIALS:_____ RATER:_____

**INTEROCEPTIVE EXPOSURE: SESSION FOUR**

1. ______Did the therapist weigh the patient?
2. ______Did the therapist review the previous week?
   1. IE challenge food tasks completed during week
   2. Attempts at counter-conditioning during week?
3. ______Did the therapist provide psycho-education on weekly skill: willful discomfort and idea of accepting discomfort/pain as necessary part of life and viewing it as something to be valued
4. ______Did the therapist complete the finger-trap exercise?
5. ______Did the therapist provide the shake?
6. ______Did the therapist provide a competent execution of exposure task via one of the following:
   1. prompting patient to increase disgust consistently throughout exposure task
   2. eliciting self-reported disgust by patient
   3. using motivational strategies to encourage patient to increase intake of shake, thereby achieving a higher level of disgust
7. ______Did the therapist encourage non-judgmental labeling of somatic symptoms during the IE exercise?
8. ______Did therapist encourage parents to adopt role as ‘coach’ during the IE exercise?
9. ______Did the therapist encourage patient to utilize weekly skill (willful discomfort) the IE exercise?
10. ______Did the therapist review the session-skill and how to apply it to IE challenge food tasks throughout upcoming week?
11. *______Did parents encourage/push child to drink more of shake during the IE exercise?
12. _____Did therapist meet alone w/parents for 10 mins to problem-solve upcoming week?
13. *____Did parents express lack of confidence in ability to implement tx? (“I can’t do this”)

ID#:_____ SESSION#:_____ LENGTH:_____ THERAPIST INITIALS:_____ RATER:_____

**INTEROCEPTIVE EXPOSURE: SESSION FIVE**

1. ______Did the therapist weigh the patient?
2. ______Did the therapist review the previous week?
   1. IE challenge food tasks completed during week
   2. Attempts at counter-conditioning during week?
3. ______Did the therapist provide psycho-education on weekly skill: positive coping and the idea of doing something to be valued despite feeling bad?
4. ______Did the therapist complete the short-term positive-coping activities exercise?
5. ______Did the therapist provide the shake?
6. ______Did the therapist provide a competent execution of exposure task via one of the following:
   1. prompting patient to increase disgust consistently throughout exposure task
   2. eliciting self-reported disgust by patient
   3. using motivational strategies to encourage patient to increase intake of shake, thereby achieving a higher level of disgust
7. ______Did the therapist encourage non-judgmental labeling of somatic symptoms during the IE exercise?
8. ______Did the therapist encourage parents to adopt role as ‘coach’ during IE exercise?
9. ______Did the therapist encourage patient to utilize the weekly skill (positive coping) during the IE exercise?
10. ______Did the therapist review the session-skill and how to apply it to IE challenge food tasks throughout upcoming week?
11. *______Did parents encourage/push child to drink more of shake during the IE exercise?
12. _____Did therapist meet alone w/parents for 10 mins to problem-solve upcoming week?
13. *____Did parents express lack of confidence in ability to implement tx? (“I can’t do this”)

ID#:_____ SESSION#:_____ LENGTH:_____ THERAPIST INITIALS:_____ RATER:_____

**INTEROCEPTIVE EXPOSURE: SESSION SIX**

1. ______Did the therapist weigh the patient?
2. ______Did the therapist review the previous week?
   1. IE challenge food tasks completed during week?
   2. Attempts at counter-conditioning during week?
3. ______Did the therapist provide psycho-education on weekly skill: acceptance and the idea of acknowledging the current situation and learning to live with it in life in a valued and productive way?
4. ______Did the therapist complete the acceptance metaphor exercises?
5. ______Did the therapist provide the shake?
6. ______Did the therapist provide a competent execution of exposure task via one of the following:
   1. prompting patient to increase disgust consistently throughout exposure task
   2. eliciting self-reported disgust by patient
   3. using motivational strategies to encourage patient to increase intake of shake, thereby achieving a higher level of disgust
7. ______Did the therapist encourage non-judgmental labeling of somatic symptoms during the IE exercise?
8. ______Did therapist encourage parents to adopt role as ‘coach’ during the IE exercise?
9. ______Did the therapist encourage patient to utilize the weekly skill (acceptance) during the IE exercise?
10. *______Did parents encourage/push child to drink more of shake during the IE exercise?
11. ______Did the therapist summarize the main points of treatment and review upcoming sessions following the end of the intervention?
12. *____Did parents express lack of confidence in ability to implement tx? (“I can’t do this”)
